# Micropapillary bladder cancer: an added indication to prophylactic urethrectomy

**DOI:** 10.1093/jscr/rjab501

**Published:** 2021-11-11

**Authors:** Vahid Mehrnoush, Asmaa Ismail, Ahmed Zakaria, Hazem Elmansy, Walid Shahrour, Owen Prowse, Ahmed Kotb

**Affiliations:** Department of Urology, Northern Ontario School of Medicine, TBRHSC, 980 Oliver Road, Thunder Bay, Ontario, P7B 6V4, Canada; Department of Urology, Northern Ontario School of Medicine, TBRHSC, 980 Oliver Road, Thunder Bay, Ontario, P7B 6V4, Canada; Department of Urology, Northern Ontario School of Medicine, TBRHSC, 980 Oliver Road, Thunder Bay, Ontario, P7B 6V4, Canada; Department of Urology, Northern Ontario School of Medicine, TBRHSC, 980 Oliver Road, Thunder Bay, Ontario, P7B 6V4, Canada; Department of Urology, Northern Ontario School of Medicine, TBRHSC, 980 Oliver Road, Thunder Bay, Ontario, P7B 6V4, Canada; Department of Urology, Northern Ontario School of Medicine, TBRHSC, 980 Oliver Road, Thunder Bay, Ontario, P7B 6V4, Canada; Department of Urology, Northern Ontario School of Medicine, TBRHSC, 980 Oliver Road, Thunder Bay, Ontario, P7B 6V4, Canada

## Abstract

There is little research on the role of urethrectomy during cystectomy in patients with micropapillary bladder cancer (MPBC). We present two cases of MPBC cystectomy and suggest that urethrectomy be performed concurrently as a preventive measure. The first case involved a woman who had a mixed solid and papillary bladder tumour. An anterior pelvic exenteration was performed as well as a total urethrectomy. The T4a micropapillary variant tumour was confirmed by pathology. The second case involved a man with T1 MPBC who was treated with a BCG induction course. A recurrent muscle-invasive MPBC was discovered during follow-up. During the radical cystoprostatectomy, the urethra was spared. T2 MPBC was discovered through pathology. He had a urethrectomy 6 months later due to urethral bleeding, and the pathology revealed micropapillary cancer of the urethra.

## INTRODUCTION

Micropapillary bladder cancer (MPBC) is a rare variant of urothelial carcinoma. Available reports are either case reports or short case series, not enabling specific guidelines to tackle this aggressive pathology. A survey distributed among members of society of urologic oncology confirmed no consensus on the proper management of these patients, although most members agreed on early cystectomy for these patients, even when presented as T1 disease [1]. A study from the national bladder cancer database showed poor prognosis for patients with MPBC regardless of the treatment modality and found no survival benefits for neoadjuvant chemotherapy [2]. Willis *et al*. studied the efficacy of intravesical BCG for patients with T1 MPBC. They studied 40 patients and detected 75% and 45% for recurrence and progression, respectively [3].

Prophylactic urethrectomy at time of radical cystectomy is debatable and not routinely done by most urologic oncologists. A recent study (2021) found a survival benefit for prophylactic nephrectomy to patients that had multiple bladder tumours or concomitant carcinoma *in situ* at time of the transurethral resection [4]. Older studies found no significant survival benefits for prophylactic urethrectomy [5]. No study looked specifically for the role of urethrectomy at time of cystectomy for patients with MPBC.

The aim of our study was to present two cases that underwent cystectomy for MPBC and advocate for prophylactic urethrectomy for these cases, based on our outcomes.

## CASES

### Case 1

**Figure 1:**
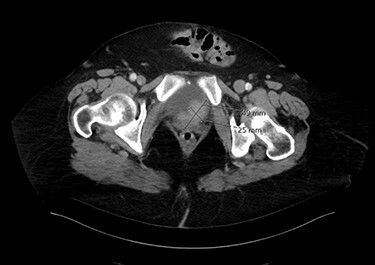
An axial image of the bladder tumour.

Eighty-year-old lady presented to the urology care with persistent dysuria and recurrent UTI. Cystoscopy showed mixed solid and papillary bladder tumour in the trigonal area and grossly normal bladder neck. Endoscopic resection confirmed muscle-invasive MPBC. Anterior pelvic exenteration with total urethrectomy was done. Pathology confirmed T4a micropapillary variant, infiltrating the vaginal wall and urethra. There was positive perivesical lymph nodes as well as positive bilateral iliac pelvic lymph nodes. Figure 1 shows an axial image of the bladder tumour.

### Case 2

**Figure 2:**
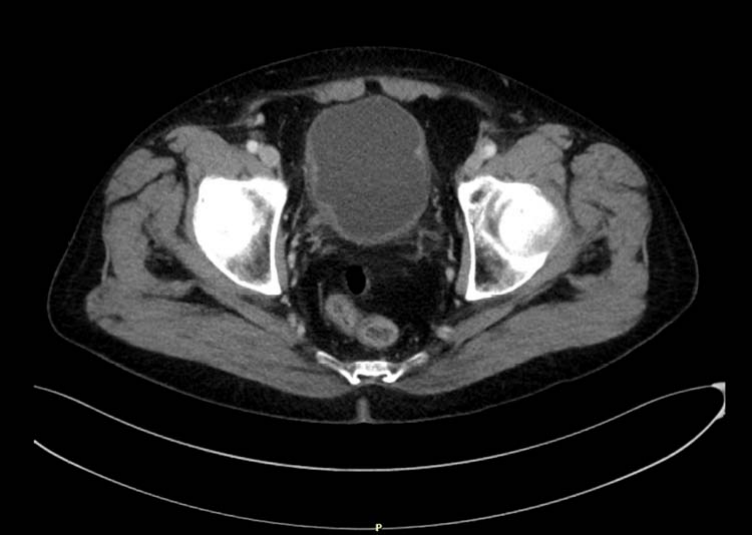
An axial image of the bladder tumour.

Seventy-year-old gentleman presented with an attack of gross hematuria and computed tomography scan showing irregularly thickened bladder wall. Endoscopic resection confirmed T1 MPBC. Induction course of BCG was provided. Cystoscopy showed small mass recurrence and pathology was muscle-invasive MPBC. Radical cystoprostatectomy sparing the urethra was done. Pathology was T2 MPBC with negative surgical margins and negative for lymph nodes invasion. He developed bleeding per urethra 6 months later and urethrectomy was done showing micropapillary cancer involving the urethra. Figure 2 shows an axial image of the bladder tumour.

## DISCUSSION

MPBC is a very aggressive variant of bladder cancer. Most of the currently available few publications confirm cystectomy being the main way to treat that cancer. Recent studies showed neoadjuvant chemotherapy did not offer a survival advantage for this aggressive variant [6, 7]. Both of our cases were discussed in our multi-disciplinary team meetings and there was an agreement to avoid neoadjuvant chemotherapy and to proceed with upfront cystectomy. No single publication, however; was found discussing the role of prophylactic urethrectomy for patients with MPBC.

The beneficial role for prophylactic urethrectomy in patients with urothelial carcinoma is very debatable and most of us do not offer it because of low rate of urethral recurrence. Hakozaki *et al.* [4] found that prophylactic urethrectomy was the only independent predictor of overall survival in patients with multiple bladder tumours and/or concomitant carcinoma *in situ* that were treated without neoadjuvant chemotherapy. Van Poppel and Thuer [8] recommended the use of prostatic urethral involvement in men and bladder neck involvement in women as the only indication for prophylactic urethrectomy. Canadian urology association guidelines recommend urethrectomy for men with high-grade or invasive urethral disease distal to prostatic urethra, a positive urethral margin or suspected prostatic stromal involvement and for women with bladder neck involvement [9]. EAU–ESMO consensus statements included a recommendation for early and frequent urethral surveillance for patients with MPBC in whom the tumour was multifocal, has associated carcinoma *in situ* or involving the prostatic urethra [10].

We are presenting two cases; one case for a lady that had the tumour trigonal but not invading the bladder neck. Urethrectomy was done and was found involved with cancer. The other patient was a man that did not have prostatic urethral involvement or concomitant carcinoma *in situ*. Prophylactic urethrectomy was not done. Surgical margin was negative but developed a urethral recurrence within 6 months requiring perineal urethrectomy.

While it is hard to make a strong recommendation out of two case reports, we think MPBC may be considered as a significant risk for urethral involvement and/or recurrence and may be a factor to trigger prophylactic urethrectomy at time of the radical cystectomy.

## CONCLUSION

Prophylactic urethrectomy may be considered at the time of radical cystectomy, for patients with MPBC, regardless of other risk factors for urethral involvement.

## CONFLICT OF INTEREST STATEMENT

None declared.
